# Diagnostic performance of deep learning for infectious keratitis: a systematic review and meta-analysis

**DOI:** 10.1016/j.eclinm.2024.102887

**Published:** 2024-10-18

**Authors:** Zun Zheng Ong, Youssef Sadek, Riaz Qureshi, Su-Hsun Liu, Tianjing Li, Xiaoxuan Liu, Yemisi Takwoingi, Viknesh Sounderajah, Hutan Ashrafian, Daniel S.W. Ting, Jodhbir S. Mehta, Saaeha Rauz, Dalia G. Said, Harminder S. Dua, Matthew J. Burton, Darren S.J. Ting

**Affiliations:** a Birmingham and Midland Eye Centre, Sandwell and West Birmingham NHS Trust, Birmingham, UK; b Birmingham Medical School, College of Medicine and Health, University of Birmingham, UK; c Department of Ophthalmology and Department of Epidemiology, University of Colorado Anschutz Medical Campus, Aurora, CO, USA; d Department of Inflammation and Ageing, College of Medicine and Health, University of Birmingham, UK; e Department of Ophthalmology, University Hospitals Birmingham NHS Foundation Trust, Birmingham, UK; f Health Data Research UK, London, UK; g Department of Applied Health Sciences, University of Birmingham, Birmingham, UK; h Institute of Global Health Innovation, Imperial College London, London, UK; i Singapore National Eye Centre, Singapore Eye Research Institute, Singapore; j Ophthalmology and Visual Sciences Academic Clinical Program, Duke-NUS Medical School, Singapore; k Academic Ophthalmology, School of Medicine, University of Nottingham, Nottingham, UK; l Department of Ophthalmology, Queen’s Medical Centre, Nottingham, UK; m International Centre for Eye Health, London School of Hygiene and Tropical Medicine, London, UK; n National Institute for Health Research (NIHR) Biomedical Research Centre, Moorfields Eye Hospital NHS Foundation Trust and UCL Institute of Ophthalmology, London, UK

**Keywords:** Artificial intelligence, Corneal infection, Corneal ulcer, Deep learning, Infectious keratitis, Microbial keratitis

## Abstract

**Background:**

Infectious keratitis (IK) is the leading cause of corneal blindness globally. Deep learning (DL) is an emerging tool for medical diagnosis, though its value in IK is unclear. We aimed to assess the diagnostic accuracy of DL for IK and its comparative accuracy with ophthalmologists.

**Methods:**

In this systematic review and meta-analysis, we searched EMBASE, MEDLINE, and clinical registries for studies related to DL for IK published between 1974 and July 16, 2024. We performed meta-analyses using bivariate models to estimate summary sensitivities and specificities. This systematic review was registered with PROSPERO (CRD42022348596).

**Findings:**

Of 963 studies identified, 35 studies (136,401 corneal images from >56,011 patients) were included. Most studies had low risk of bias (68.6%) and low applicability concern (91.4%) in all domains of QUADAS-2, except the index test domain. Against the reference standard of expert consensus and/or microbiological results (seven external validation studies; 10,675 images), the summary estimates (95% CI) for sensitivity and specificity of DL for IK were 86.2% (71.6–93.9) and 96.3% (91.5–98.5). From 28 internal validation studies (16,059 images), summary estimates for sensitivity and specificity were 91.6% (86.8–94.8) and 90.7% (84.8–94.5). Based on seven studies (4007 images), DL and ophthalmologists had comparable summary sensitivity [89.2% (82.2–93.6) versus 82.2% (71.5–89.5); P = 0.20] and specificity [(93.2% (85.5–97.0) versus 89.6% (78.8–95.2); P = 0.45].

**Interpretation:**

DL models may have good diagnostic accuracy for IK and comparable performance to ophthalmologists. These findings should be interpreted with caution due to the image-based analysis that did not account for potential correlation within individuals, relatively homogeneous population studies, lack of pre-specification of DL thresholds, and limited external validation. Future studies should improve their reporting, data diversity, external validation, transparency, and explainability to increase the reliability and generalisability of DL models for clinical deployment.

**Funding:**

10.13039/100000002NIH, 10.13039/100010269Wellcome Trust, MRC, 10.13039/501100000615Fight for Sight, 10.13039/501100014631BHP, and 10.13039/100010399ESCRS.

**Research in context**

**Evidence before this study**

Infectious keratitis (IK), commonly known as corneal infection, is the leading cause of corneal blindness globally. Timely diagnosis is imperative for achieving favourable clinical outcomes. However, current diagnostic approach is challenged by low microbiological culture yield, long turnaround time for culture results, and need for clinical expertise, which is particularly lacking in low- and middle-income countries (LMICs). All these issues underscore the need for innovative solutions to improve IK diagnosis. Deep learning (DL) – a subset of artificial intelligence – has demonstrated considerable promise in enabling medical diagnoses, though its value in IK remains unclear. We conducted a systematic search across EMBASE (OVID), MEDLINE (OVID), DANS EASY Archive, and trial registries, to identify studies investigating the diagnostic accuracy of DL models for IK (based on any type of corneal imaging) published from 1974 until July 16, 2024. We identified one recent systematic review which assessed the diagnostic accuracy of DL in IK. However, this review was limited by a small sample size (n = 11 studies), inclusion of slit-lamp/anterior segment photograph-based studies only, invalid statistical methods, and lack of meta-analytic comparison between DL models and ophthalmologists.

**Added value of this study**

This review, which adheres to Cochrane methods, represents the most comprehensive examination of DL models for diagnosing IK to date (based 35 studies with 136,401 corneal images from >56,011 patients). In addition, to our knowledge, this is the first and only review that has systematically evaluated the performance of DL models, based on both internal and external validation studies, and compared accuracy with that of ophthalmologists. Our meta-analyses (based on images as the unit of analysis) found DL may have good diagnostic accuracy for IK, particularly in diagnosing the presence of any IK, and to a lesser extent, in differentiating the underlying causes of IK. For IK, DL had comparable sensitivity and specificity with those of ophthalmologists, potentially supporting the use of DL models in real-world settings. Methodological quality assessment using the QUADAS-2 tool showed most studies had low risk of bias (68.6%) and low applicability concern (91.4%) in terms of patient selection, reference standard and flow and timing. However, there was high risk of bias and high applicability concern in the index test domain due to lack of threshold pre-specification and limited external validation. This is likely to overestimate diagnostic accuracy and affect the generalisability of our findings.

**Implications of all the available evidence**

DL models may have good diagnostic accuracy for IK and comparable performance to ophthalmologists, highlighting its potential clinical value as a medical aid in real-world settings. However, diagnostic accuracy may be unduly precise due to using multiple images from an individual without accounting for potential correlation within individuals, relatively homogeneous population studies, lack of threshold pre-specification, and limited external validation. Future studies need to improve their reporting, data diversity, external validation, transparency, and explainability to increase the reliability and generalisability of DL models.

## Introduction

Infectious keratitis (IK), commonly known as corneal infection, is the leading cause of corneal blindness globally.^1^^,^^2^ Once considered a “silent epidemic” in low- and middle-income countries (LMICs), IK has resulted in ∼5 million cases of blindness worldwide and accounts for ∼2 million cases of monocular blindness annually.^2^^,^^3^ The annual incidence of IK is disproportionately higher in LMICs (113–799 per 100,000 people) than in high-income countries (HICs; 2.5–40.3 per 100,000 people),^2^^,^^4^, ^5^, ^6^ primarily due to limited access to eye care and increased trauma, amongst other risk factors. A recent meta-analysis estimated that the global incidence of fungal keratitis alone (excluding other causes of IK) is projected to exceed one million cases annually, predominantly affecting Asian and African populations.^7^ In view of its significant global public health burden, a consortium-led proposal has called for the designation of IK as a neglected tropical disease, aiming to draw concerted and sustained global effort to tackle IK in LMICs.^8^

IK can be caused by a wide array of pathogens, including bacteria, fungi, protozoa, and viruses. Patients afflicted by IK often experience profound ocular discomfort and vision impairment, with some losing the entire eye due to intractable infection.^9^, ^10^, ^11^, ^12^, ^13^ Timely and accurate diagnosis is crucial for achieving a good clinical outcome in IK, though this is currently challenged by the variable low yield and relatively high costs of conventional microbiological culture, long turnaround time for positive results, poorly differentiated clinical features (among different causes of IK), reliance on clinical expertise/equipment, and delay in seeking medical attention.^14^^,^^15^ All these issues highlight an unmet need for innovative solutions to improve the diagnosis of IK.

In recent years, there has been a surge of interest in integrating artificial intelligence (AI) into clinical medicine, including the field of infectious diseases, ranging from diagnosis, risk stratification, disease outbreak surveillance, and antimicrobial drug discovery/development.^16^, ^17^, ^18^ Deep learning (DL), a subset of AI, has shown significant potential in aiding automated medical diagnostics, clinical prioritisation, decision-making processes, and streamlining healthcare workflows in both HICs and LMICs.^19^, ^20^, ^21^, ^22^ While DL has shown considerable promise as a diagnostic tool for several ophthalmic conditions,^19^^,^^20^^,^^23^ its clinical potential for diagnosing IK remains to be fully elucidated.^24^^,^^25^

This systematic review aimed to evaluate the diagnostic accuracy of DL models for IK using corneal imaging, compare their accuracy with that of ophthalmologists, and investigate methodological issues for improving future research and potential clinical deployment.

## Methods

This systematic review and meta-analysis was conducted in accordance with recommendations in the Cochrane Handbook for Systematic Reviews of Diagnostic Test Accuracy,^26^ and reporting followed the Preferred Reporting Items for Systematic Review and Meta-Analysis for Diagnostic Test Accuracy Studies (PRISMA-DTA).^27^ The systematic review protocol was registered with PROSPERO (CRD42022348596) and published.^28^

### Search strategy and selection criteria

We performed a comprehensive search of bibliographic databases, including EMBASE (OVID), MEDLINE (OVID), IEEE Xplore, and DANS EASY Archive, and trial registries, including the Cochrane CENTRAL, ISRCTN registry (www.isrctn.com/), US NIH Ongoing Trials Register (https://www.clinicaltrials.gov/), and WHO International Clinical Trials Registry Platform (ICTRP). The search was first performed on May 8, 2022, and last updated on July 16, 2024. We also manually searched the bibliographies and citations of the included studies to identify any additional potentially relevant studies. There was no restriction on study design, publication year, or language for the search. The search strategy, including keywords and index terms, was adapted to each information source. An example of the search strategy is provided in Supplementary Table S1.

Two reviewers (ZZO and YS) independently screened the abstracts and assessed the full-text of potentially eligible studies, with disagreements adjudicated by a senior author (DSJT). We included all diagnostic accuracy studies, including clinical trials, cross-sectional studies, prospective and retrospective cohort studies, and case–control studies, that examined the accuracy of DL models for diagnosing any type of IK, encompassing bacterial, fungal, *Acanthamoeba*, and/or viral keratitis. We included only studies that used corneal imaging, such as slit-lamp/anterior segment photography (ASP), *in vivo* confocal microscopy (IVCM), anterior segment optical coherence tomography, and/or corneal topography/tomography. Depending on the study design and target condition(s), the reference standard was either expert consensus, microbiological results, and/or treatment response, or a composite reference standard. Exclusion criteria included reviews, case reports, studies that did not use any corneal imaging, or those that focused on image segmentation instead of disease classification. There was no restriction on patient age, gender, ethnicity, study location, or sample size.

### Data analysis

Two reviewer authors (ZZO and YS) independently extracted the data separately using a pre-defined data extraction sheet. Any disagreement was adjudicated by a senior author (DSJT). Study authors were contacted to request additional data or clarification where necessary. We included all eligible studies for qualitative assessment, and where possible, we constructed 2 × 2 contingency tables for calculation of sensitivity and specificity. We extracted data from both internal and external validation studies of DL models as well as the performance of ophthalmologists, with the intent of meta-analysing these three sets of data separately. Internal validation refers to the evaluation of DL models based on the dataset from the same data source used to develop the model, whereas external validation involves testing the developed DL models using an independent dataset (derived from a different source/population). We used image as the unit of analysis as this was most commonly used and reported in DL studies. We recognise images from the same eye and same person are likely to be correlated, but we did not have individual participant data to allow us to account for the potential correlation. Therefore, our analysis using aggregate data might lead to unduly precise estimates (i.e., narrower confidence intervals) of the diagnostic accuracy of DL models. Where multiple accuracy estimates were reported for DL in a study (e.g. results generated from different algorithms for the same dataset), we only included the best performing DL model (based on the best sensitivity) in the meta-analyses as we were interested in study-level outcomes.

We presented summary estimates of sensitivity and specificity with 95% confidence intervals (CIs) from each included primary study on forest plots. We generated summary receiver operating characteristic (SROC) plots and 95% confidence/prediction regions around the point estimates for each target disease to visually assess heterogeneity as recommended by the Cochrane Handbook for Systematic Reviews of Diagnostic Accuracy.^26^ The I^2^ statistic (commonly used in intervention meta-analysis reviews) was not used in this review as it does not account for heterogeneity due to threshold effects induced by the relationship between sensitivity and specificity and is also susceptible to precision of the included studies. In addition, the mean and variance of proportions such as sensitivity and specificity are related, and such mean-variance relationships can lead to biased I^2^ estimates because of ignoring variability in the within-study variance across studies.^26^ We expected heterogeneity in the types of DL systems and algorithms used across studies and considered all to be acceptable as our review aimed to assess the accuracy of any DL system for corneal imaging. In view of the anticipated between-study heterogeneity, we used random-effects models for all meta-analyses. To jointly synthesise sensitivities and specificities in each meta-analysis, we fitted a bivariate model. We performed analyses using the user written command metandi and the ‘meqrlogit’ command in Stata 15. We investigated the effect of imaging type on sensitivity and specificity by adding covariate terms to the bivariate model (bivariate meta-regression). We used bivariate meta-regression to also compare the accuracy of DL models and ophthalmologists. We computed absolute differences in sensitivity and specificity post-estimation of the bivariate model parameters using the nlcom command with P values for the differences from Wald tests.

We performed subgroup analyses by: (1) classification of the target disease (e.g. distinguishing IK from healthy eyes/non-IK corneal pathologies or differentiating the underlying causes of IK); and (2) corneal imaging (e.g. ASP versus IVCM). For studies which included both classifications of the target disease, our meta-analysis focussed primarily on the DL ability to differentiate the underlying causes of IK as it is expected to provide more clinical value.

Two independent reviewer authors (ZZO and YS) critically appraised the included studies for methodological rigor using the Quality Assessment of Diagnostic Accuracy Studies-2 (QUADAS-2) tool to examine risk of bias in four domains, including patient selection, index test, reference standard, and flow and timing, as well as applicability in the first three domains.^29^

### Role of the funding source

The funders of this study had no role in study design, data collection, data analysis/interpretation, or writing of the report.

## Results

**Fig. 1:**
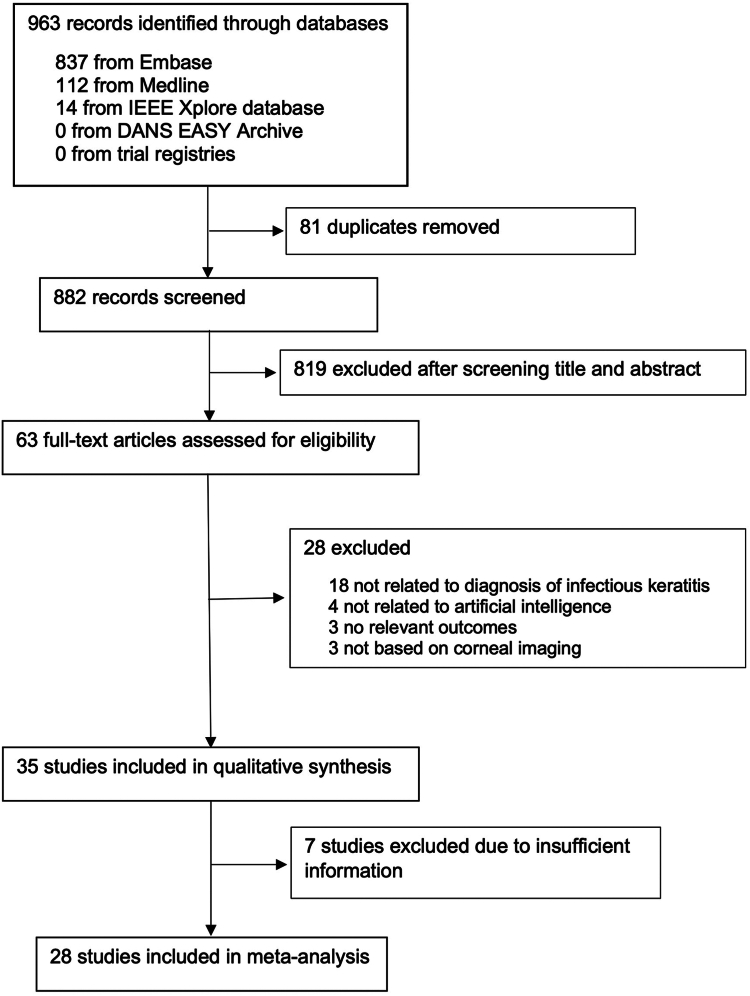
PRISMA flow chart of study selection.

Our initial search identified 963 articles, of which 882 studies (after de-duplication) were screened and 63 full-text articles were assessed for eligibility (Fig. 1). After excluding 28 ineligible studies, we included 35 studies (at least 56,011 patients, with 136,401 corneal images) published between 2018 and 2024.^30^, ^31^, ^32^, ^33^, ^34^, ^35^, ^36^, ^37^, ^38^, ^39^, ^40^, ^41^, ^42^, ^43^, ^44^, ^45^, ^46^, ^47^, ^48^, ^49^, ^50^, ^51^, ^52^, ^53^, ^54^, ^55^, ^56^, ^57^, ^58^, ^59^, ^60^, ^61^, ^62^, ^63^, ^64^ Ten studies^30^^,^^31^^,^^35^^,^^37^^,^^44^^,^^47^^,^^49^, ^50^, ^51^^,^^63^ reported only the number of images but not the patients. The 35 studies were conducted in eight countries, with China being the commonest location (21, 60.0%). Key characteristics of the included studies are summarised in Table 1.

**Table 1:** Summary of key characteristics of included studies.

Authors (Year)	Country	Inclusion criteria	Exclusion criteria	No. of patients	No. of images	Mean age (SD; range) years	Study design	Target condition	Reference standard^a^	Imaging modality
Cai et al. (2021)^30^	China	Various corneal pathologies, including IK	Duplicated, incorrect magnification images, and photographs with a lack of clarity were excluded	NR	112	NR	Cross-sectional	Corneal ulcer versus other pathology	Expert consensus	ASP
Essalat et al. (2023)^31^	Iran	IK	Poor quality images	NR	4001	NR	Cross-sectional	FK and AK versus other IK and normal	Microbiology, expert consensus	IVCM
Ghosh et al. (2022)^32^	Thailand	BK and FK	Low quality images, mixed infections	194	2167	NR	Cross-sectional	BK versus FK	Microbiology, treatment response	ASP
Gu et al. (2020)^33^	China	Various corneal pathologies, including IK	NR	5835	5835	NR	Cross-sectional	IK versus other corneal pathologies	Expert consensus	ASP
Hong et al. (2021)^34^	China	Various corneal pathologies, including IK	Dilated pupil images	NR	1098	NR	Cross-sectional	IK versus other pathology and normal	Expert Consensus	ASP
Hou et al. (2021)^35^	China	FK	NR	NR	1870	NR	Cross-sectional	FK versus normal	Expert consensus	IVCM
Hu et al. (2023)^36^	China	IK	Inconclusive diagnosis, mixed infections, other corneal diseases, poor quality	744	2757	NR	Cross-sectional	IK versus Normal and differentiating IK causes (BK,VK,FK)	Expert consensus, microbiology	ASP
Huang et al. (2022)^37^	China	FK	Mixed infections, ocular surface disease, thyroid eye disease, viral keratitis, poor image quality	NR	2157	NR	Cross-sectional	Fusarium FK versus non-Fusarium FK	Expert consensus, microbiology	ASP
Hung et al. (2021)^38^	Taiwan	BK and FK	Mixed infections, poor quality images, history of other corneal diseases	580	1330	55.4 ± 20.2	Cross-sectional	BK versus FK	Expert consensus, microbiology	ASP
Kogachi et al. (2023)^39^	India	BK and FK	Missing results on one or more microbiologic tests.	886	1970	NR	Cross-sectional	Morphological differences between images of microbiologically positive and negative corneal ulcers	Expert consensus, microbiology (culture and smear)	ASP
Koyama et al. (2021)^40^	Japan	IK	Mixed infections	362	4306	59.4 ± 21.8	Cross-sectional	Differentiate IK causes (BK, FK, AK, and HSK)	Expert consensus, microbiology	ASP
Kuo et al. (2020)^41^	Taiwan	IK	Mixed infection, no initial photo	288	288	NR	Cross-sectional	FK versus other IK	Microbiology	ASP
Kuo et al. (2021)^42^	Taiwan	IK	Mixed infections or no consensus	1512	1512	NR	Cross-sectional	BK versus other IK	Expert consensus, microbiology	ASP
Kuo et al. (2022)^43^	Taiwan	BK	Contaminated, mixed infections	929	929	NR	Cross-sectional	Pseudomonas BK versus non-Pseudomonas BK	Expert consensus, microbiology (smear and culture)	ASP
Li et al. (2020)^44^	China	Various types of corneal pathologies (including IK)	Lack of clarity, low contrast or duplications	NR	2437	43.8	Cross-sectional	IK versus other corneal pathologies and cataract	Expert consensus	ASP
Li et al. (2021)^45^	China	Various types of corneal pathologies, including IK	Poor-quality and unreadable images	7988	13,557	NEH dataset (41.6/4–98), ZEH dataset (39.2/10–83), JEH dataset (42.3/8–96), NOC dataset (45.7/5–89), Smartphone dataset (44.3/5–90)	Cross-sectional	IK versus other corneal pathologies and normal	Expert consensus, microbiology	ASP
Li et al. (2022)^46^	China	BK, FK and HSK	NR	519	1886	NR	Cross-sectional	IK versus normal and differentiating IK causes (BK, FK, and HSK)	Expert consensus, microbiology (culture), IVCM	ASP
Li et al. (2023)^47^	China	FK	NR	NR	423	NR	Cross-sectional	FK versus other IK	Expert consensus, microbiology	ASP
Li et al. (2024)^48^	China	IK	Poor quality images, lack of diagnostic certainty	10,369	23,055	53.6	Cross-sectional	Differentiate BK,FK,VK, AK and NIK	Expert consensus, microbiology	ASP
Liang et al. (2023)^49^	China	FK	NR	NR	7278	NR	Cross-sectional	FK with hyphae versus non-hyphae	Expert consensus	IVCM
Liu et al. (2020)^50^	China	FK	NR	NR	1870	NR	Cross-sectional	FK versus normal	Microbiology	IVCM
Lv et al. (2020)^51^	China	FK	Poor image quality, interfering conditions	NR	2623	NR	Cross-sectional	FK versus other IK	Expert consensus, microbiology	IVCM
Natarajan et al. (2022)^52^	India	HSK	Resolving or scarred infections or microbiologically negative cases. Mixed infections	285	307	NR	Cross-sectional	Stromal viral keratitis (HSK) versus other IK	Microbiology (culture or PCR)	ASP
Redd et al. (2022)^53^	India	BK and FK	Culture-negative or polymicrobial infections	980	980	NR	Cross-sectional	BK versus FK	Microbiology (culture or smear)	ASP
Soleimani et al. (2023)^54^	Iran	BK and FK	Mixed infections, had corneal graft procedures, ocular surface conditions, poor quality image	977	9329	NR	Cross-sectional	BK versus FK versus normal	Microbiology (culture)	ASP
Tang et al. (2023)^55^	China	FK	Blurry images, and images without hyphae structure were excluded	NR	3364	NR	Cross-sectional	Fusarium FK versus other FK, and aspergillus FK versus other FK	Microbiology (culture)	IVCM
Tiwari et al. (2022)^56^	India and USA	BK and FK	Cases with no definite diagnosis	1124	1124	NR	Cross-sectional	Differentiate corneal ulcer and scar (other pathology)	Microbiology (culture)	ASP
Ueno et al. (2024)^57^	Japan	Various corneal diseases including IK	Poor quality images	6443	6443	NR	Cross-sectional	IK versus other corneal diseases	Expert consensus, microbiology	ASP
Wang et al. (2021)^58^	China	BK, FK and HSK	NR	3320	6073	Normal: 55.0, BK: 53.1, FK: 60.6, HSK: 52.2	Cross-sectional	Differentiate IK causes (BK, FK, HSK)	Expert consensus, microbiology	ASP
Wei et al. (2023)^59^	China	BK, FK, and AK	Mixed infections, poor images, history of ocular surface diseases	1496	1916	50.4	Cross-sectional	Differentiate FK from other IK	Expert consensus, microbiology (culture or scraping)	ASP
Won et al. (2023)^60^	Korea	IK (BK and FK)	Viral or Acanthamoeba keratitis were excluded	107	684	NR	Cross-sectional	BK versus FK	Microbiology (culture and scraping)	ASP
Wu et al. (2018)^61^	China	BK and FK	Mixed infections, Age > 65 years or multi-comorbidities	79	56	48.0 ± 6.3	Cross-sectional	BK versus FK	Microbiology (smear and culture) and IVCM	IVCM
Wu et al. (2023)^62^	China	BK and FK	Mixed infections, corneal perforation, poor quality images, other corneal diseases	352	704	53.6 ± 11.5	Cross-sectional	FK versus BK	Expert consensus, microbiology (smear/culture)	ASP
Xu et al. (2021)^63^	China	BK and FK	Poor-quality images	NR	3177	NR	Cross-sectional	FK versus other IK	Expert consensus, microbiology	IVCM
Zhang et al. (2022)^64^	China	All types of IK	Mixed infections, poor images, history of other ocular inflammation	4283	5030	NR	Cross-sectional	Differentiate IK causes (BK, FK, HSK, AK)	Expert consensus, microbiology (smear/culture)	ASP

Authors (Year)	Type of internal validation	External validation	Training/validation/testing ratio^b^	AI algorithms	Diagnostic accuracy	Performance for ophthalmologists
Cai et al. (2021)^30^	Random split sampling	No	70:10:20	CNN: Residual Network, Inception, DenseNet	Sens: 64.2% (ResNet), 75.0% (InceptionV3), 60.7% (DenseNet)	NR
Essalat et al. (2023)^31^	Random split sampling with 5-fold cross-validation	No	75:25	Densenet161	Sens: 99.5% (healthy), 91.4% (AK), 97.0% (FK), 88.8% (non-specific keratitis), 94.8% (average) Spec: 98.8% (healthy), 98.3% (AK), 96.4% (FK), 98.1% (non-specific keratitis), 97.8% (average)	NR
Ghosh et al. (2022)^32^	Random split sample validation	No	85:5:10	Ensemble	AUC: 0.904 Sens: 77.0% (81.0–83.0) PPV: 91.0% (87.0–95.0)	NR
Gu et al. (2020)^33^	Random split sampling	Yes	80:20	CNN: Inception-v3	AUC: 0.930 (0.904–0.952)	NR
Hong et al. (2021)^34^	Random split sampling with 5-fold cross-validation	Yes	NR	CNN: Inception-v3 based	AUC: 0.950 Sens: 92.0%	NR
Hou et al. (2021)^35^	Random split sampling	No	70:30	CNN: AlexNet, ZFNet, VGG16	AUC: 1.000 (VGG16) Sens: 99.3% (VGG16) Spec: 99.2% (VGG16)	NR
Hu et al. (2023)^36^	Random split sampling	No	BK: 65:20:15 other groups: 70:10:20	CNN: VGG16, ResNet34, InceptionV4, DenseNet121, EffecientNetV2-M. Transformer: ViT-Base	AUC: 0.830 (VGG16), 0.820 (Resnet34), 0.860 (InceptionV4), 0.810 (Densenet121), 0.820 (Vit-Base), 0.850 (EffecientNetV2-M)	AUC: 0.890–0.970 (normal), 0.750–0.780 (VK), 0.740–0.720 (FK), 0.660–0.610 (BK)
Huang et al. (2022)^37^	NR	No	64:16:20	Inception ResNet v2	AUC: 0.785 (0.742–0.828) (original), 0.876 (0.843–0.909) (enhanced) Sens: 72.0% (original), 83.1% (enhanced) Spec: 71.6% (original), 76.6% (enhanced)	NR
Hung et al. (2021)^38^	Random split sampling with five-fold cross validation	No	66:17:17	DenseNet161	AUC: 0.850 Sens: 65.8% (41.5–65.8) Spec: 87.3% (86.0–95.3)	NR
Kogachi et al. (2023)^39^	NR	NR	80:10:10	MobileNetV2, DenseNet201	AUC: Culture results only: 0.480 (0.400–0.570) (DenseNet) 0.520 (0.440–0.600) (MobileNet) Culture and smear results: 0.560 (0.440–0.670) (DenseNet) 0.510 (0.380–0.650) (MobileNet)	NR
Koyama et al. (2021)^40^	Split sample validation with K-fold validation	Yes	80:20	InceptionResNetV2	AUC: 0.979 (AK), 0.907 (BK), 0.950 (FK), 0.923 (HSK)	AUC (AI versus clinicians): 0.820 versus 0.580 (BK), 0.840 versus 0.590 (AK), 0.780 versus 0.520 (FK), 0.730 versus 0.590 (HSK)
Kuo et al. (2020)^41^	Random split sampling with 5-fold cross-validation	No	80:20	CNN: DenseNet	AUC: 0.650 Sens: 71.1% (62.1–78.6) Spec: 68.4% (61.1–74.9)	Non-corneal ophthalmologists: Sens: 51.8% (42.7–60.7) Spec: 77.2% (70.9–83.3) Corneal specialists: Sens: 71.9% (63.1–79.4) Spec: 78.5% (72.0–84.1)
Kuo et al. (2021)^42^	Random split sampling with 5-fold cross-validation	No	80:20	CNN: SE-ResNet	AUC: 0.752 Sens: 82.4% (74.4–90.2) Spec: 54.7% (47.0−62.4)	NR
Kuo et al. (2022)^43^	Fivefold cross-validation, random split sampling	No	80:20	ResNet50,ResNext50, DenseNet121, SE-ResNet50, EfficientNet B0, EfficientNet B1, EfficientNet B2, EfficientNet B3, Ensemble model (BE2, BE3, BE4, BE5)	AUC: 0.760 (EfficientNet B2), 0.770 (BE4) Sens: 81.1% (76.3–85.8) (EfficientNet B2), 79.6% (69.0–90.3) (BE4) Spec: 51.5% (47.1–55.8) (EfficientNet B2), 57.2% (48.6–65.9) (BE4)	NR
Li et al. (2020)^44^	Fourfold cross-validation, stratified random sampling	Yes	NR	CNN: ResNet	Sens: 91.5% Spec: 93.1%	ACC: 68.0%–96.0%
Li et al. (2021)^45^	Random split sample validation	Yes	70:15:15	CNN: DenseNet121, Inception-v3, ResNet50	AUC: DenseNet121 (0.998) Sens: 97.7% (96.4–99.1) Spec: 98.2% (97.1–99.4)	ACC: 95.2%–98.3% (cornea specialist with 3 years of experience), 96.6%–98.6% (cornea specialist with 6 years of experience)
Li et al. (2022)^46^	Random split sampling, 5-fold cross-validation	No	NR	CAA-Net	AUC: 0.840 (average), 0.990 (normal), 0.810 (VK), 0.820 (FK), 0.750 (BK) Sens: 66.1% (average) Spec: 66.9% (average)	NR
Li et al. (2023)^47^	Random split sampling, 5-fold cross-validation	No	80:20	Model 1: DenseNet 121, mobienet_v2, squeezentet1_0 models, (LASSO) model, MLP classifier Model 2: Automatic segmentation and DL model	AUC: 0.839 (0.751–0.927) (Model 1); 0.925 (0.869–0.981) (Model 2) Sens: 86.1% (Model 1); 90.5% (Model 2) Spec: 76.2% (Model 1); 85.7% (Model 2)	NR
Li et al. (2024)^48^	Random split sampling	Yes	70:15:15	CNN (Densenet121, Inceptionresnetv2, Swin-transformer, DeepIK)	DeepIK (best-performing) AUC: 0.949 (0.937–0.960) (BK); 0.970 (0.961–0.979) (FK); 0.955 (0.946–0.964) (VK); 0.994 (0.988–0.999) (AK); 0.979 (0.972–0.984) (NIK) Sens: 76.9% (71.8–82%) (BK); 79.7% (74.9–84.5%) (FK); 83.5% (80.6–86.3%) (VK); 75.0% (65.0–85.0%) (AK); 89.3% (86.7–91.9%) (NIK) Spec: 93.8% (92.5–95%) (BK); 96.6% (95.7–97.5%) (FK); 91.7% (90.10–93.3%) (VK); 99.9% (99.8–100%) (AK); 95.5% (94.4–96.7%) (NIK)	DeepIK versus Ophthal Sens: 74% versus 63% (BK) 78% versus 66% (FK) 80% versus 70.5% (VK) 66% versus 54.5% (AK) 84% versus 74.5% (NIK) DeepIK versus Ophthal Spec: 88.5% versus 89.4% (BK) 94.5% versus 92.5% (FK) 94% versus 85% (VK) 98.5% versus 99.1% (AK) 95% versus 91.1% (NIK)
Liang et al. (2023)^49^	Random split sampling	No	80:20	SACNN	AUC: 0.993 Sens: 97.0% Spec: 98.5%	NR
Liu et al. (2020)^50^	Random split sampling	No	91:9	CNN: AlexNet, VGG16	Sens: 99.9% (Novel AlexNet), 99.8% (Novel VGG16) Spec: 100% (Novel AlexNet), 100% (Novel VGG16)	NR
Lv et al. (2020)^51^	Random split sample validation with 5-fold cross-validation	Yes	NR	CNN: ResNet	AUC: 0.988 (0.976–0.991) (no diabetes), 0.977 (0.976–0.991) (diabetes) Sens: 91.9% (no diabetes); 82.6% (diabetes) Spec: 98.3% (no diabetes); 98.9% (diabetes)	NR
Natarajan et al. (2022)^52^	Random split sampling	No	87:13	DenseNet-201	AUC: 0.730 (0.568–0.892) Sens: 69.6% Spec: 76.5%	NR
Redd et al. (2022)^53^	Stratified random sampling	Yes	75:10:15	CNN: MobileNetV2, DenseNet201, Ensemble method	AUC: 0.860 (0.780–0.930) (MobileNetV2), 0.840 (0.760–0.920) (DenseNet201), 0.840 (0.760–0.920) (Ensemble method)	AUC: 0.790 (0.690–0.890)
Soleimani et al. (2023)^54^	Random split sample validation with 5-fold cross-validation	No	72:8:20	CNN: Adam	AUC: 0.999 (healthy); 0.960 (BK versus FK); 0.990 (filamentous versus yeast) Sens: 99.3% (healthy); 84.0% (BK versus FK); 77.5% (filamentous versus yeast) Spec: 99.2% (healthy); 84.0% (BK versus FK); 76.6% (filamentous versus yeast)	NR
Tang et al. (2023)^55^	Random split sampling	No	90:10	DT classifier model, DL classifier model	AUC: 0.786 (0.736–0.837) (DT Fusarium), 0.887 (0.853–0.922) (DL Fusarium), 0.737 (0.687–0.784) (DT Aspergillus), 0.828 (0.782–0.866) (DL Aspergillus)	NR
Tiwari et al. (2022)^56^	Random split sample validation	Yes	60:20:20	CNN: VGG16	AUC: 0.973 (MUTT trials), 0.947 (Byers) Sens: 93.5% (89.1–97.9) (MUTT trials), 78.2% (67.3–89.1) (Byers) Spec: 84.4% (79.42–89.42) (MUTT trials), 91.3% (85.8–96.8) (Byers)	NR
Ueno et al. (2024)^57^	Random split sampling	Yes	86:14	YOLO v3, v5 and retinanet	YOLO v5 AUC: 0.996 (0.978–0.997) (IK) Sens: 88.7% (86.3–90.8%) (IK) Spec: 97.7% (97.3–98.2%) (IK)	Yes
Wang et al. (2021)^58^	Random split sample validation	Yes	80:10:10	CNN: Inception, Residual Network, DenseNet	AUC: 0.959 (0.943–0.975) (InceptionV3), 0.952 (0.934–0.970) (ResNet50), 0.961 (0.945–0.977) (DenseNet121)	AUC: 0.852 (0.823–0.881)
Wei et al. (2023)^59^	Random split sampling	Yes	70:30	Internal validation: Binary logistic regression, random forest classification, decision tree classification External validation: Binary logistic regression	Internal validation: AUC: 0.859–0.916 Sens: 94.8%–98.0% Spec: 73.7%–88.3% External validation (binary logistic regression): AUC: 0.903 (0.808–0.998) Sens: 90.7% (77.4–100) Spec: 89.9% (75.0–100)	Sens: 69.1% (46.7–76.7) Spec: 71.7% (52.0–83.3)
Won et al. (2023)^60^	NR	Yes	87:13	ResNEt-50 Proposed method	Sens: 75.0% (ResNEt-50); 86.4% (Proposed method) Spec: 87.0% (ResNEt-50), 89.1% (Proposed method)	NR
Wu et al. (2018)^61^	NR	No	NR	Support Vector Machine	AUC: 0.946 Sens: 89.3% Spec: 95.7%	NR
Wu et al. (2023)^62^	Random split sampling	No	64:16:20	CNN (Resnet50, Resnet 152, Densenet 121, Densenet169)	AUC: 0.88 (Resnet152) Sens: 92.0% (Resnet152) Spec: 83.0% (Resnet 152)	NR
Xu et al. (2021)^63^	Stratified random sampling	No	NR	CNN: Residual learning network-101	AUC: 0.983 Sens: 93.6% Spec: 98.2%	ACC: 89.4% (8.88–89.9) (without AI assistance), 93.3% (92.7–93.9) (with AI assistance), 94.2% (93.3–95.1) (with XAI assistance)
Zhang et al. (2022)^64^	Random split methods, validated by 10-fold cross-validation.	Yes	90:10	Combination model: KeratitisNet (combination of ResNext101_32 × 16 d and DenseNet169)	AUC: 0.860 (BK), 0.910 (FK), 0.960 (AK), 0.980 (HSK)	NR

ACC = Accuracy; AI = Artificial intelligence; AK = Acanthamoeba keratitis; ASP = Anterior segment photography; AUC = Area under the ROC curve; BK = Bacterial keratitis; CNN = Convolutional neural network; FK = Fungal keratitis; HSK = Herpes simplex keratitis; IK = Infectious keratitis; IVCM = *In vivo* confocal microscopy; NIK = Non-infectious keratitis; NR = Not reported; Sens = Sensitivity; Spec = Specificity; VK = viral keratitis; XAI = Explainable artificial intelligence.

a Expert consensus = Diagnosis of IK is made by one or more ophthalmologists.

b Some studies only have training and validation dataset.

Of the 35 studies, ten (28.6%) and seven (20.0%) studies focused on distinguishing IK from healthy corneas^33^, ^34^, ^35^, ^36^^,^^45^^,^^46^^,^^48^^,^^50^^,^^54^^,^^58^ and from non-IK corneal pathologies,^30^^,^^33^^,^^34^^,^^44^^,^^45^^,^^56^^,^^57^ respectively. Twenty-six (74.3%) studies examined the performance of DL models in differentiating the underlying causes of IK, including six (17.1%) studies on various IK such as bacterial, fungal, *Acanthamoeba*, and/or viral keratitis^36^^,^^40^^,^^46^^,^^48^^,^^58^^,^^64^ seven (20.0%) on bacterial keratitis versus fungal keratitis,^32^^,^^38^^,^^53^^,^^54^^,^^60^, ^61^, ^62^ five (14.3%) on fungal keratitis versus other causes of IK,^41^^,^^47^^,^^51^^,^^59^^,^^63^ three (8.6%) on fungal keratitis alone,^37^^,^^49^^,^^55^ one (2.9%) on fungal keratitis versus *Acanthamoeba* keratitis,^31^ one (2.9%) on bacterial keratitis versus other causes of IK,^42^ one (2.9%) on viral keratitis versus other causes of IK,^52^ one (2.9%) on bacterial keratitis alone,^43^ and one (2.9%) on microbiological-positive versus microbiological-negative bacterial/fungal keratitis.^39^ Six (17.1%) studies focused on multiple classifications.^33^^,^^34^^,^^36^^,^^45^^,^^46^^,^^64^ Twenty-six (74.3%) studies used ASP,^30^^,^^32^, ^33^, ^34^^,^^36^^,^^38^, ^39^, ^40^, ^41^, ^42^, ^43^, ^44^, ^45^, ^46^, ^47^, ^48^^,^^52^, ^53^, ^54^^,^^56^, ^57^, ^58^, ^59^, ^60^^,^^62^^,^^64^ while nine (25.7%) used IVCM images.^31^^,^^35^^,^^37^^,^^49^, ^50^, ^51^^,^^55^^,^^61^^,^^63^ Of the 26 ASP-based studies, all (100%) used slit lamp/digital cameras to acquire corneal images,^30^^,^^32^, ^33^, ^34^^,^^36^^,^^38^, ^39^, ^40^, ^41^, ^42^, ^43^, ^44^, ^45^, ^46^, ^47^, ^48^^,^^52^, ^53^, ^54^^,^^56^, ^57^, ^58^, ^59^, ^60^^,^^62^^,^^64^ while two studies also used smartphone-captured images as one of the external validation sets.^45^^,^^58^ Among IVCM-based studies, eight (88.9%) employed the Heidelberg HRT III RCM^35^^,^^37^^,^^49^, ^50^, ^51^^,^^55^^,^^61^^,^^63^ and one (11.1%) used the NIDEK confoscan 3.0.^31^

All 35 studies were cross-sectional studies; 27 (77.1%) used retrospective data,^30^, ^31^, ^32^^,^^35^^,^^36^^,^^38^^,^^40^, ^41^, ^42^, ^43^^,^^45^, ^46^, ^47^^,^^49^, ^50^, ^51^, ^52^, ^53^, ^54^, ^55^, ^56^^,^^58^^,^^60^^,^^62^, ^63^, ^64^ six (17.1%) used both prospective and retrospective data,^33^^,^^34^^,^^44^^,^^48^^,^^57^^,^^59^ and two (5.7%) studies used prospective data.^39^^,^^61^ Most studies (30, 85.7%) excluded mixed infections,^31^, ^32^, ^33^^,^^35^, ^36^, ^37^, ^38^, ^39^, ^40^, ^41^, ^42^, ^43^, ^44^^,^^46^^,^^47^^,^^49^, ^50^, ^51^, ^52^, ^53^, ^54^, ^55^, ^56^^,^^58^, ^59^, ^60^, ^61^, ^62^, ^63^, ^64^ 19 (54.3%) excluded low-quality images,^30^, ^31^, ^32^^,^^36^, ^37^, ^38^^,^^44^^,^^45^^,^^48^^,^^49^^,^^51^^,^^53^, ^54^, ^55^, ^56^, ^57^^,^^62^, ^63^, ^64^ and six (17.1%) did not provide details regarding their exclusion criteria.^33^^,^^35^^,^^46^^,^^49^^,^^50^^,^^58^ Various reference standards were used: 19 (54.3%) studies used expert consensus and microbiological confirmation,^31^^,^^36^, ^37^, ^38^, ^39^, ^40^^,^^42^^,^^43^^,^^45^, ^46^, ^47^, ^48^^,^^51^^,^^57^, ^58^, ^59^^,^^62^, ^63^, ^64^ nine (25.7%) used microbiological confirmation (based on smear, culture, and/or PCR testing) alone,^41^^,^^50^^,^^52^, ^53^, ^54^, ^55^, ^56^^,^^60^^,^^61^ six (17.1%) used expert consensus only,^30^^,^^33^, ^34^, ^35^^,^^44^^,^^49^ and one (2.9%) used microbiological confirmation and treatment response.^32^ Most studies (31, 88.6%) used convolutional neural networks (CNNs) as the primary DL models.^30^, ^31^, ^32^, ^33^, ^34^, ^35^, ^36^, ^37^, ^38^^,^^40^, ^41^, ^42^, ^43^, ^44^, ^45^, ^46^, ^47^, ^48^, ^49^, ^50^, ^51^, ^52^, ^53^, ^54^^,^^56^^,^^58^^,^^60^^,^^62^, ^63^, ^64^ Fourteen (40.0%) studies used external validation,^33^^,^^34^^,^^40^^,^^44^^,^^45^^,^^48^^,^^51^^,^^53^^,^^56^, ^57^, ^58^, ^59^, ^60^^,^^64^ and 14 (40.0%) compared the diagnostic accuracy of DL models with ophthalmologists,^33^^,^^34^^,^^36^^,^^40^^,^^41^^,^^44^^,^^45^^,^^48^^,^^53^^,^^57^, ^58^, ^59^^,^^63^^,^^64^ though only seven (20.0%) studies provided sufficient 2 × 2 data for head-to-head meta-analysis (see below). The most common data split for training and validation/testing was 80:20 [n = 9 (25.7%) studies].^33^^,^^39^, ^40^, ^41^, ^42^, ^43^^,^^47^^,^^49^^,^^58^

**Supplementary Fig. S1:**
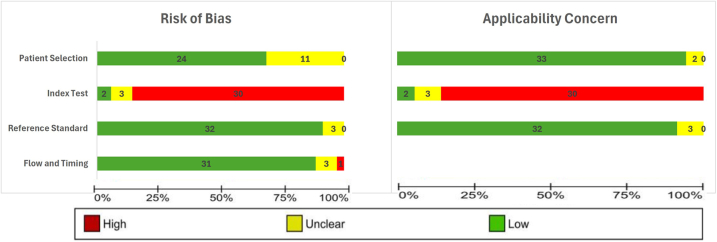
Supplementary Fig. S1. Summary of QUADAS-2 study assessment for risk of bias and applicability concern.

Most studies (68.6%) were judged to have low risk of bias in all three domains, namely patient selection, reference standard, and flow and timing domains, but high risk of bias in the index test domain (Supplementary Fig. S1 and Table S2). Eleven (31.4%) studies^30^^,^^35^^,^^44^^,^^46^^,^^47^^,^^49^, ^50^, ^51^^,^^54^^,^^58^^,^^62^ were deemed to have an unclear risk of bias, due to unclear source/process of patient selection. Thirty (85.7%) studies were at high risk of bias in the index test domain due to the lack of pre-specified threshold. Three (8.6%) studies^35^^,^^47^^,^^50^ had an unclear risk of bias in the reference standard domain due to uncertainties in the reference standard used. Four (11.4%) studies^32^^,^^35^^,^^58^^,^^59^ had a high/unclear risk in flow and timing domain due to the potential inconsistency of reference standard used. For applicability, most studies had low concern regarding patient selection (33, 94.2%) and reference standard (32, 91.4%) but high concern in the index test domain (30, 85.7%) due to potential overestimation of the diagnostic accuracy of DL because of the lack of threshold pre-specification.

**Fig. 2:**
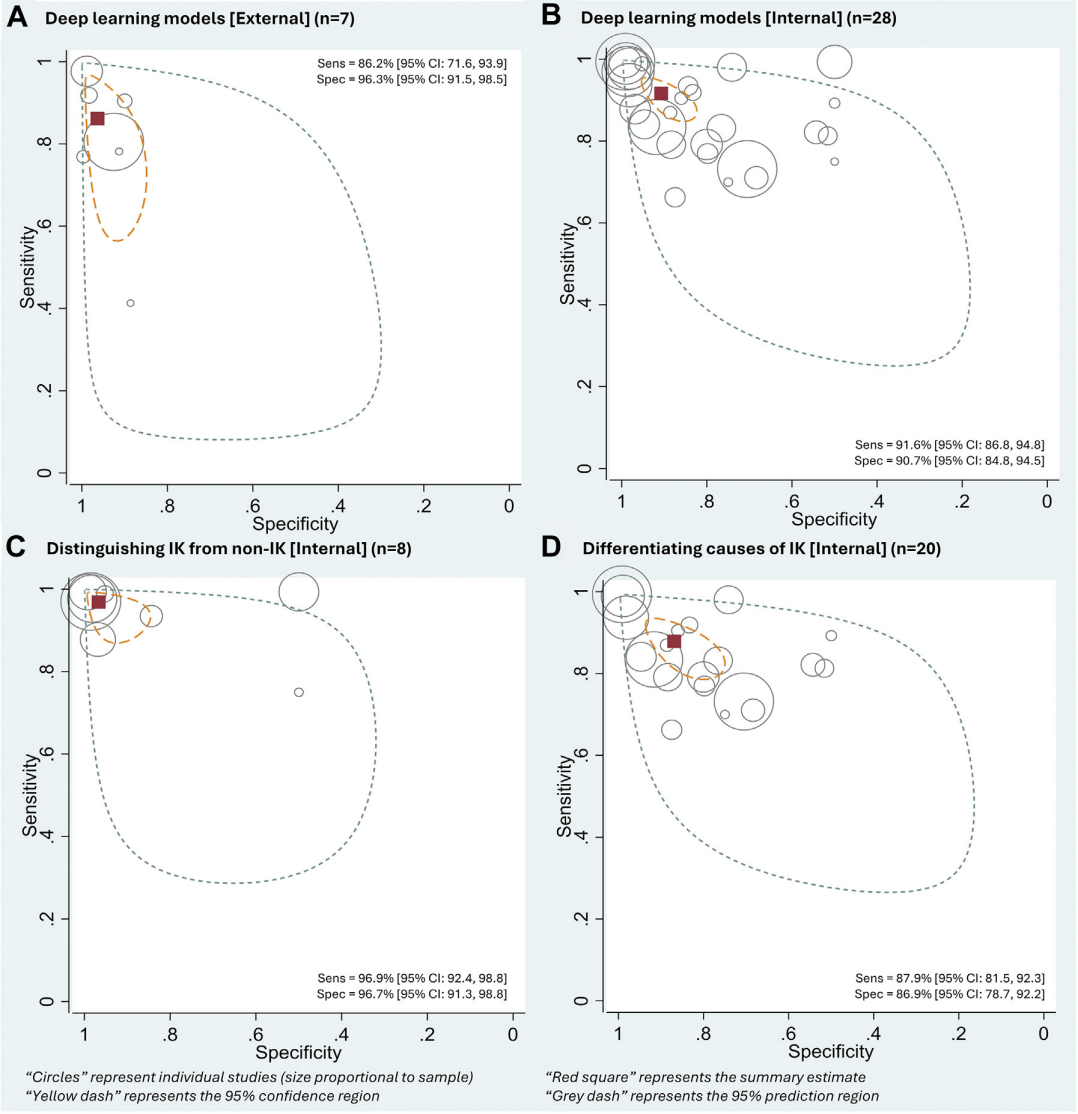
Summary receiver operating characteristic (SROC) plots for: (A) Diagnostic accuracy of deep learning (DL) models for any IK (external validation; seven studies, 10,675 images); (B) Diagnostic accuracy of DL for infectious keratitis (IK) (internal validation; 28 studies, 16,059 images); (C) Diagnostic accuracy of DL for distinguishing IK from healthy corneas/non-IK corneal pathologies (internal validation; eight studies, 4479 images), and (D) Diagnostic accuracy of DL for differentiating causes of IK (internal validation; 20 studies, 11,580 images).

**Fig. 3:**
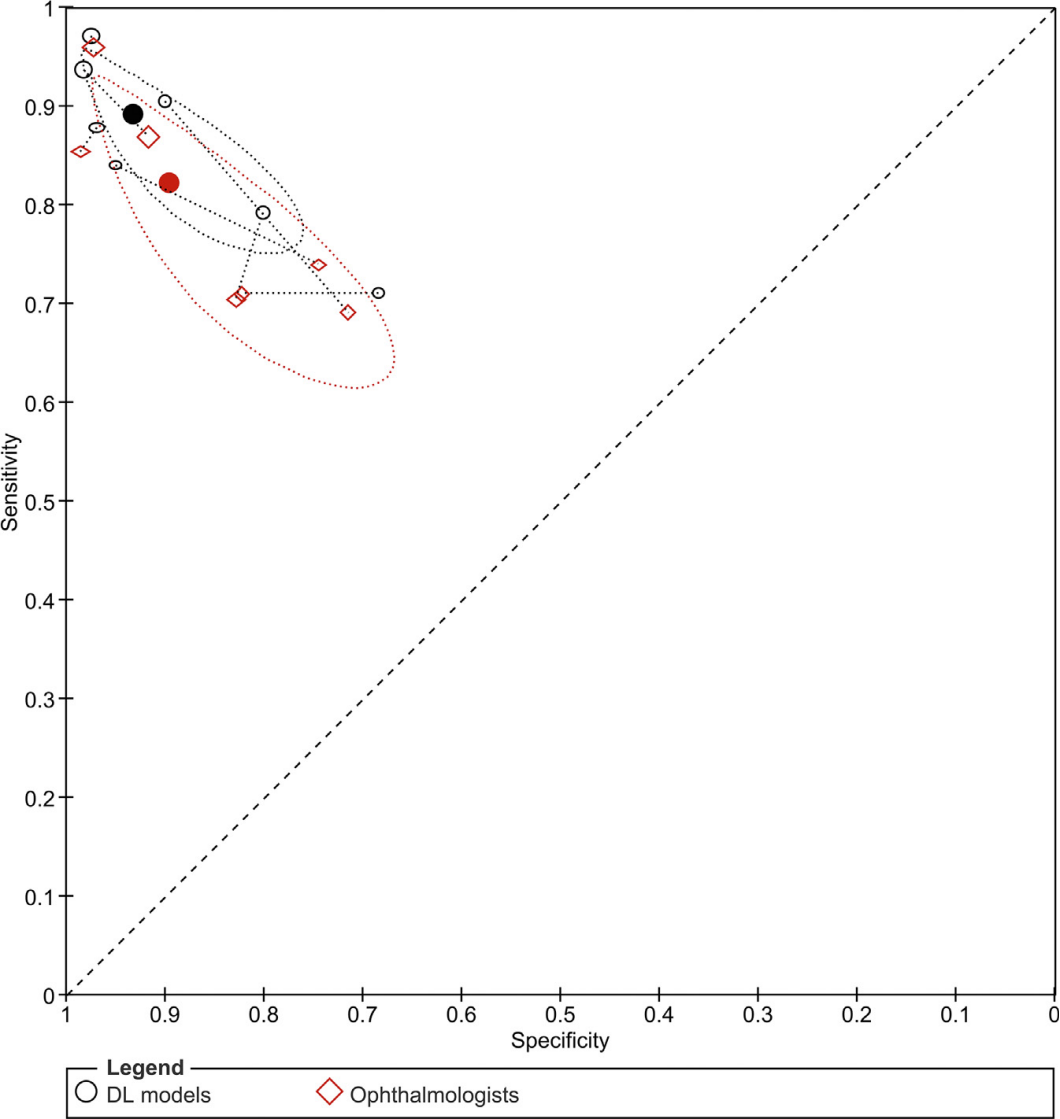
Summary receiver operating characteristic (SROC) plot of deep learning (DL) models versus ophthalmologists (based on seven studies, 4007 images). The hollow symbols are the study points for each index test with dotted lines connecting the pair of points from each study. The study points have been scaled by sample size to reflect the precision of the estimates of sensitivity and specificity from the studies. The solid circles are the summary points representing the summary sensitivities and specificities. Each summary point is surrounded by a 95% confidence region which illustrates the uncertainty around the estimates of sensitivity and specificity.

**Supplementary Fig. S2:**
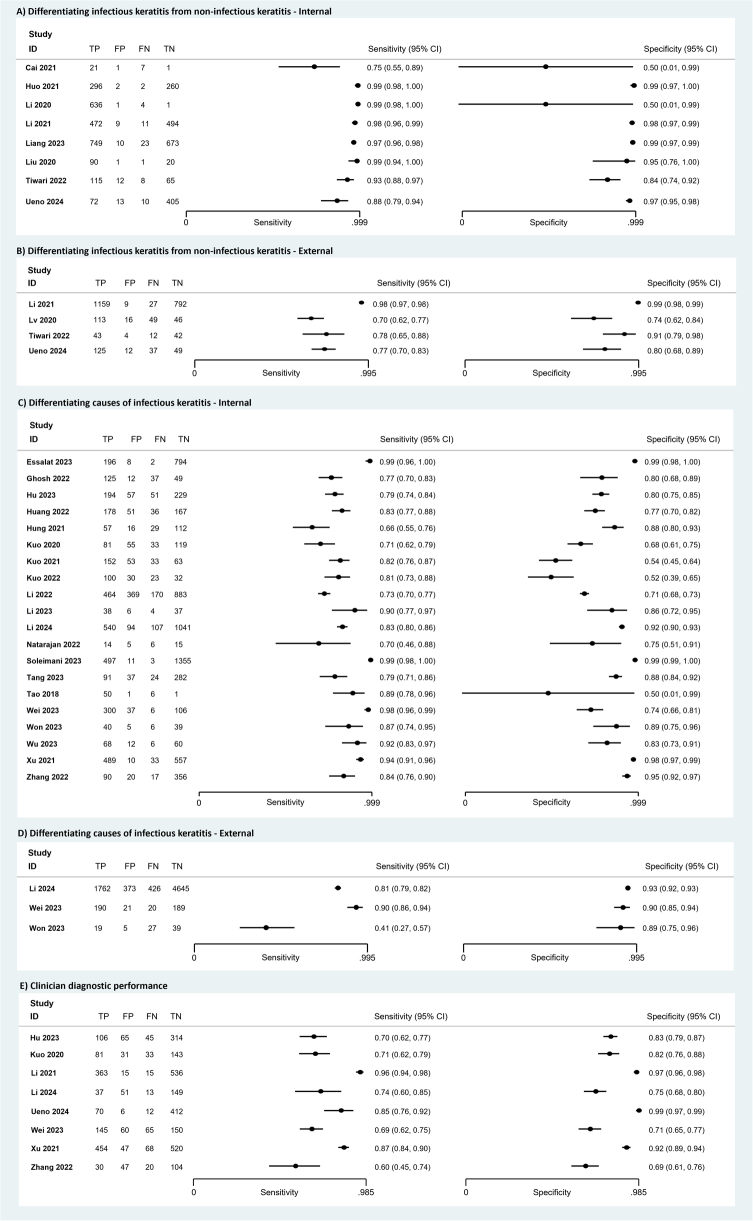
Supplementary Fig. S2. Forest plots showing the sensitivity and specificity of each included study.

Based on external validation data (seven studies, 10,675 images) the sensitivity and specificity were 86.2% (71.6–93.9) and 96.3% (91.5–98.5) (Table 2 and Fig. 2). For internal validation data (28 studies, 16,059 images), the sensitivity and specificity of DL for diagnosis of IK were 91.6% (86.8–94.8) and 90.7% (84.8–94.5). Subgroup analyses of the two target disease classifications were performed using internal validation data only as there were insufficient data from external validation studies. Based on eight studies (4479 images)^30^^,^^35^^,^^44^^,^^45^^,^^49^^,^^50^^,^^56^^,^^57^ for distinguishing IK from healthy corneas/non-IK corneal pathologies, the sensitivity and specificity were 96.9% (92.4–98.8) and 96.7% (91.3–98.8). For differentiating the causes of IK (20 studies, 11,580 images),^36^, ^37^, ^38^^,^^41^, ^42^, ^43^^,^^46^^,^^48^^,^^52^^,^^54^^,^^55^^,^^59^^,^^61^, ^62^, ^63^ DL had a sensitivity of 87.9% (81.5–92.3) and 86.9% (78.7–92.2). Based on the seven studies (four internal and three external validation studies, 4007 images) that compared the accuracy of DL models with ophthalmologists (using the same reference standard and corneal images in both groups),^36^^,^^41^^,^^45^^,^^48^^,^^57^^,^^59^^,^^63^ DL models had higher sensitivity [89.2% (82.2–93.6) versus 82.2% (71.5–89.5); P = 0.20] and specificity [(93.2% (85.5–97.0) versus 89.6% (78.8–95.2); P = 0.45] than ophthalmologists, though not statistically significant (Table 2 and Fig. 3). The absolute differences in sensitivity and specificity were 7.0% (−3.6 to 17.5) and 3.7% (−5.8 to 13.1). Diagnostic accuracy of all included studies is detailed in Supplementary Fig. S2 and Table S3.

**Table 2:** Overview of meta-analytic results of the performance of deep learning (DL) and clinicians for infectious keratitis (IK).

Model (N = # studies | n = # images)	Sensitivity (95% CI)		P-value	Specificity (95% CI)		P-value
1. DL performance (External validation)						
Overall (N = 7 | n = 10,675)	86.2%	(71.6–93.9)	–	96.3%	(91.5–98.5)	–
2. DL performance (Internal validation)						
Overall (N = 28 | n = 16,059)	91.6%	(86.8–94.8)	–	90.7%	(84.8–94.5)	–
3. Distinguishing IK from healthy eyes/non-IK corneal pathologies (Internal validation)^a^						
Overall (N = 8 | n = 4479)	96.9%	(92.4–98.8)	–	96.7%	(91.3–98.8)	–
ASP (N = 5 | n = 2354)	94.6%	(84.9–98.2)		94.7%	(78.7–98.8)	
IVCM (N = 3 | n = 2125)	98.8%	(94.3–99.7)		98.6%	(91.8–99.8)	
4. Differentiating causes of IK (Internal validation)^b^						
Overall (N = 20 | n = 11,580)	87.9%	(81.5–92.3)	0.27	86.9%	(78.7–92.2)	0.06
ASP (N = 15 | n = 8569)	86.2%	(78.2–91.7)		83.6%	(73.3–90.5)	
IVCM (N = 5 | n = 3011)	91.8%	(80.8–96.8)		94.0%	(83.5–98.0)	
5. DL versus clinicians (Studies that performed direct comparison)^b^						
DL (N = 7 | n = 4007)	89.2%	(82.2–93.6)	0.20	93.2%	(85.5–97.0)	0.45
Clinician (N = 7 | n = 4007)	82.2%	(71.5–89.5)		89.6%	(78.8–95.2)	

ASP = Anterior segment photography; IVCM = *In vivo* confocal microscopy.

a Statistical comparison between ASP and IVCM groups was not possible due to small number of studies.

b Statistical comparison made between ASP and IVCM groups or between DL and ophthalmologists using bivariate meta-regression with Wald tests. P-value of <0.05 is considered statistically significant.

## Discussion

Previous systematic reviews and meta-analyses have reported the diagnostic accuracy of DL in medical imaging.^22^^,^^65^ However, their broad scope (all types of medical imaging for any medical condition) limited the interpretation of the role of DL for a specific medical condition. To our knowledge, this study represents the most up-to-date and comprehensive systematic review and meta-analysis specifically evaluating the diagnostic accuracy of DL in IK. Based on 35 studies with ≥56,011 patients (136,401 corneal images), DL appears to have good diagnostic accuracy for IK, including its ability to distinguish IK from healthy eyes or non-IK corneal pathologies, and to a lesser extent, to differentiate the underlying causes of IK. When compared to ophthalmologists, DL models exhibit comparable diagnostic accuracy in IK, supporting its potential use in real-world settings. Based on our systematic literature search, we identified only one published systematic review that had similarly evaluated the diagnostic accuracy of DL in IK.^66^ However, the review was limited by several critical aspects, including the relatively small number of included studies (n = 11 studies), the inclusion of slit-lamp/ASP-based studies only, lack of distinction/analysis in the performance among internal and external validation studies, and ophthalmologists, and most importantly, the unconventional/inappropriate meta-analytic approach that was adopted (i.e. directly deriving the summary results based on the reported AUC without constructing the 2 × 2 tables), which questions the validity of their findings.

IK is primarily diagnosed using clinical criteria (usually with slit lamp examination) supplemented by microbiological investigations and/or imaging tests such as slit-lamp/ASP, IVCM, and other modalities.^14^ However, the diagnosis of IK often requires considerable clinical expertise. Our review showed that DL models may have good diagnostic accuracy for IK. Based on ASP, DL models achieved a sensitivity of 96.9% and a specificity of 96.7% in diagnosing/distinguishing IK from healthy corneas/non-IK corneal pathologies. This finding highlights the potential of DL models to facilitate early and automated diagnosis of IK in primary care settings, providing an innovative solution to an unmet global need, particularly in LMICs where access to ophthalmologists is limited and IK is most prevalent.

Another diagnostic challenge in IK lies in the difficulty of identifying the underlying microbiological causes due to overlapping clinical signs, wide-ranging causative organisms, and variably low microbiological culture yield. A previous international survey showed that even corneal experts were only able to correctly distinguish bacterial keratitis from fungal keratitis in 65% of cases based on clinical signs alone.^67^ This challenge was further substantiated in a recent survey among 66 corneal specialists from 16 countries, where the accuracy in distinguishing bacterial and fungal keratitis was only 49–76% based on ASP alone.^68^ Significant disparities in diagnostic accuracy was noted among the corneal specialists, with specialists in India being more proficient in diagnosing fungal keratitis than those practicing outside India. This is likely due to a higher level of experience among the Indian experts in managing fungal keratitis, which is significantly more prevalent in India than other parts of the world such as the United States.^68^ Our meta-analysis showed that DL models, based on ASP, may have good diagnostic performance (86.2% sensitivity, 83.6% specificity) in differentiating the causes of IK. This indicates the potential of DL as an aid for clinical experts, particularly in identifying less frequently encountered causes of IK.

This study also included DL models that used IVCM images. IVCM is a corneal imaging tool that enables high-resolution imaging on the cellular level. It is useful for assisting the diagnosis of IK, particularly filamentous fungal keratitis and *Acanthamoeba* keratitis, where it can visualise fungal hyphae and *Acanthamoeba* cysts and/or trophozoites.^14^^,^^69^^,^^70^ However, interpretation of IVCM images requires substantial clinical expertise, a gap which can potentially be addressed by AI. Our results highlight that DL models, based on IVCM images, may accurately distinguish IK from healthy corneas/non-IK corneal pathologies (98.8% sensitivity and 98.6% specificity) as well as differentiate the underlying causes of IK (91.8% sensitivity and 94.0% specificity). Interestingly, IVCM-based DL models appear to perform better than the ASP-based DL models in differentiating the underlying causes of IK. The difference in performance may be attributable to a difference in patient selection as IVCM is usually performed when fungal, *Acanthamoeba* and/or atypical infections are suspected, whereas ASP is used to capture all types of IK. In addition, IVCM produces more consistent and high-contrast images whereas ASP is less standardised and more prone to missing subtle corneal pathologies (due to the transparent nature of the cornea). However, clinically related issues such as small field of view, highly operator-dependent (for obtaining good quality images), and limited availability of IVCM need to be considered.^14^^,^^71^^,^^72^

Although the performance of DL models appears promising in this review, it is important to contextualise the results and interpret them with care in view of the heterogeneity of the included studies. Some studies included only images with or without IK (but not other types of corneal pathologies), which means that some DL models are restricted to a particular medical classification task (i.e. distinguishing IK from healthy corneas or diagnosing a particular type of IK). That said, these DL models may still play a valuable assistive role in under-resourced regions where IK is most prevalent and clinical expertise is scarce. In addition, we performed the meta-analyses based on two broad disease classification tasks, which both demonstrated good DL diagnostic accuracy. Comparison between DL models and ophthalmologists showed comparable diagnostic accuracy, supporting the potential of DL for assisting the diagnosis of IK in real-world settings.

In terms of overall completeness and applicability of evidence, this systematic review and meta-analysis included studies spanning several countries with diverse economic backgrounds, encompassing LMICs and HICs. Notably, South Asia and East Asia, recognised for their high rates of IK, were well-represented in this study, offering valuable insights into the diverse presentations of IK.^6^^,^^7^ Various imaging modalities such as ASP and IVCM targeting various causes of IK were included, mirroring the clinical variations and complexities of IK encountered in real-world clinical settings. This broad-based approach enhances the applicability and generalisability of the findings to real-life scenarios. Another strength is that all the images used in the included studies were sourced from independent local patient cohorts in real-world clinical settings rather than relying on publicly available databases, which prevents overlap of data sources. In addition, the majority (82.9%) of the studies used microbiological confirmation (smear microscopy, culture, and/or PCR) as the reference standard or as part of the composite reference standard, which helps ensure disease verification. Although this approach is currently considered the best available reference standard, it may not capture all IK cases by definition. Future studies evaluating the role of AI in complementing the current diagnostic approach (e.g. increasing diagnostic sensitivity) would be of value. We adopted a proactive approach in ensuring methodological rigor and relevance of our review using the recommended QUADAS-2 tool while awaiting the development of QUADAS-AI tool.^73^

Several limitations are recognised in this systematic review, including selecting the best performing DL model where multiple accuracy estimates were reported and the use of images without accounting for potential correlation of images from the same patient. Previous research and our meta-analysis show that internal validation tends to overestimate diagnostic accuracy of DL models (due to overfitting), emphasising the importance of external validation for ascertaining the generalisability of DL models.^22^ Based on the seven external validation studies, we showed good diagnostic accuracy of IK (86.2% sensitivity and 96.3% specificity). Another limitation is that most of the studies lacked clarity on the reporting of patient characteristics and focused on relatively homogenous populations. The lack of diversity may potentially introduce algorithm bias and affect the generalisability and fairness of DL models, as highlighted by the recent STANDING Together initiative.^74^ The heterogeneous DL models/architectures used across different studies pose challenges in selecting the best-performing DL model. Finally, many of the studies did not address the inherent AI-related ‘black-box’ issue, which may hinder their acceptance among clinicians.^75^ This lack of transparency poses important medicolegal concerns as clinicians are ultimately responsible for their patients. Visualisation techniques such as Grad-CAM have been employed to enhance the transparency and explainability of DL models.^76^ We did not include studies that performed multimodal analysis, though only one study was identified.^62^

DL holds considerable promise for IK, with comparable diagnostic accuracy to ophthalmologists. However, future studies need to focus on improving study reporting (e.g. STARD-AI),^77^ data diversity, external validation, transparency of AI algorithms/architectures, and explainability to increase the reliability and generalisability of DL models. As the technology matures, it is anticipated that DL is likely to transform the diagnostic landscape of IK in both HICs and LMICs.

## Contributors

Conceptualisation: DSJT; Data curation: ZZO, YS, RQ, SHL, DSJT; Data analysis: RQ, YT; Data interpretation: ZZO, YS, RQ, SHL, TL, XL, YT, VS, HA, DSWT, JSM, SR, DGS, HSD, MJB, DSJT; Visualisation: ZZO, YS, RQ, SHL, TL, YT, DSJT; Writing – original draft: ZZO, YS, RQ, SHL, DSJT; Writing – review & editing: TL, XL, YT, VS, HA, DSWT, JSM, SR, DGS, HSD, MJB; Project administration: ZZO, YS, RQ, SHL, DSJT; Funding acquisition: DSJT; Supervision: DSJT. ZZO, YS, RQ, and DSJT accessed and verified the underlying data. DSJT was responsible for the decision to submit the manuscript.

## Data sharing statement

All data supporting the findings of this study are available within the paper and its Supplementary information.

## Declaration of interests

HA is the Chief Scientific Officer of Preemptive Medicine and Health, Flagship Pioneering.
