# Supplementary material for: Diagnostic performance of deep learning for infectious keratitis: a systematic review and meta-analysis
Source: eClinicalMedicine. 2024 Oct 18;77:102887. doi: 10.1016/j.eclinm.2024.102887 (PMC11513659; doi:10.1016/j.eclinm.2024.102887)
Supplement: Supplementary Table S1 [file mmc1.docx]

**Supplementary Table 1.** Search strategy for EMBASE.

| **#** | **Query** | **Results from 1974 to 17 Jul 2024** |
| --- | --- | --- |
| 1 | exp artificial intelligence/ | 109,715 |
| 2 | artificial intelligence.mp. | 93,669 |
| 3 | exp machine learning/ | 493,669 |
| 4 | machine learning.mp. | 172,793 |
| 5 | exp deep learning/ | 60,401 |
| 6 | deep learning.mp. | 83,784 |
| 7 | machine intelligence.mp. | 388 |
| 8 | exp support vector machine/ | 46,464 |
| 9 | support vector machine.mp. | 51,604 |
| 10 | computer-assisted.mp. | 1,085,980 |
| 11 | visual data exploration.mp. | 25 |
| 12 | 1 or 2 or 3 or 4 or 5 or 6 or 7 or 8 or 9 or 10 or 11 | 1,621,565 |
| 13 | exp keratitis/ | 39,379 |
| 14 | keratitis.mp. | 30,172 |
| 15 | infectious keratitis.mp. | 1,697 |
| 16 | infective keratitis.mp. | 152 |
| 17 | exp microbial keratitis/ | 9,879 |
| 18 | microbial keratitis.mp. | 2,118 |
| 19 | corneal infection.mp. | 1,179 |
| 20 | exp cornea ulcer/ | 8,847 |
| 21 | corneal ulcer.mp. | 2,968 |
| 22 | 13 or 14 or 15 or 16 or 17 or 18 or 19 or 20 or 21 | 43,088 |
| 23 | 12 and 22 | 961 |
| 24 | limit 23 to human | 837 |
