# Supplementary material for: Diagnostic performance of deep learning for infectious keratitis: a systematic review and meta-analysis
Source: eClinicalMedicine. 2024 Oct 18;77:102887. doi: 10.1016/j.eclinm.2024.102887 (PMC11513659; doi:10.1016/j.eclinm.2024.102887)
Supplement: Supplementary Table S2 [file mmc2.docx]

**Supplementary Table 2.** QUADAS study assessment for included studies related to deep learning for diagnosing infectious keratitis.

| **Study ID** | ***Applicability: “Are there concerns that the [ _____ ] does not match the review question?”*** | | | | | | | | | | | | **Domain 4 –  [ Timeline ]** | | | | |
| --- | --- | --- | --- | --- | --- | --- | --- | --- | --- | --- | --- | --- | --- | --- | --- | --- | --- |
|  | **Domain 1 –  [ Patient selection ]** | | | | | | **Domain 2 –  [ Index test ]*** | | | **Domain 3 –  [ Reference standard]** | | |  |  |  |  |  |
| ***Signaling questions*** | *Was a consecutive or random sample of eligible patients enrolled?* | *Was a case-control design avoided?* | *Did the study avoid inappropriate exclusions?* | *Did the study avoid pre-processing of the images?* | *Did the study avoid including mixed infections?* | *Overall: Could the selection of patients introduce bias?* | *Were the index test results interpreted without knowledge of the results of the reference standard?* | *If a threshold was used, was it pre-specified?* | *Overall: Could the conduct or interpretation of the index test have introduced bias?* | *Is the reference standard likely to correctly classify the target condition?* | *Were the reference standard results interpreted without knowledge of the results of the index test?* | *Overall: Could the reference standard, its conduct, or its interpretation have introduced bias?* | *Was there an appropriate interval between index tests and reference standard?* | *Did all patients receive a reference standard?* | *Did all patients receive the same reference standard?* | *Were all patients included in the analysis?* | *Overall: Could the patient flow have introduced bias?* |
| Cai 2021 | 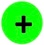 | | | | | | 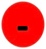 | | | 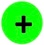 | | | Not applicable | | | | |
|  | 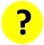 | 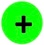 | 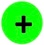 | 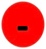 | 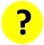 | 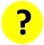 | 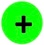 | 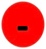 | 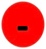 | 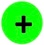 | 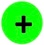 | 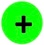 | 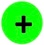 | 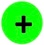 | 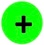 | 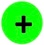 | 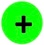 |
| Essalat 2023 | 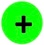 | | | | | | 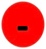 | | | 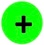 | | | Not applicable | | | | |
|  | 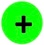 | 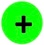 | 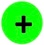 | 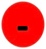 | 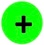 | 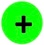 | 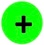 | 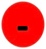 | 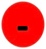 | 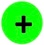 | 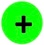 | 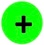 | 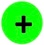 | 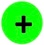 | 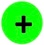 | 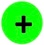 | 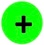 |
| Ghosh 2022 | 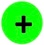 | | | | | | 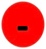 | | | 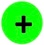 | | | Not applicable | | | | |
|  | 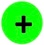 | 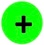 | 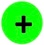 | 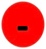 | 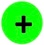 | 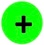 | 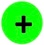 | 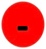 | 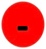 | 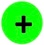 | 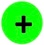 | 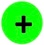 | 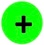 | 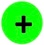 | 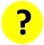 | 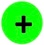 | 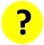 |
| Gu 2020 | 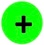 | | | | | | 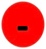 | | | 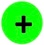 | | | Not applicable | | | | |
|  | 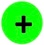 | 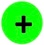 | 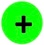 | 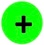 | 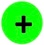 | 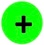 | 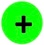 | 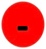 | 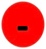 | 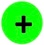 | 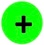 | 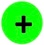 | 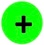 | 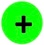 | 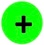 | 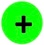 | 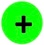 |
| Hong 2021 | 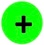 | | | | | | 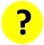 | | | 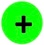 | | | Not applicable | | | | |
|  | 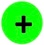 | 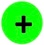 | 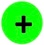 | 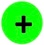 | 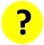 | 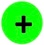 | 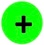 | 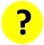 | 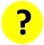 | 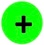 | 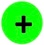 | 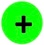 | 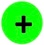 | 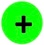 | 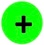 | 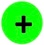 | 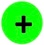 |
| Hou 2021 |  | | | | | |  | | |  | | | Not applicable | | | | |
|  |  |  |  |  |  |  |  |  |  |  |  |  |  |  |  |  |  |
| Hu 2021 |  | | | | | |  | | |  | | | Not applicable | | | | |
|  |  |  |  |  |  |  |  |  |  |  |  |  |  |  |  |  |  |
| Huang 2022 |  | | | | | |  | | |  | | | Not applicable | | | | |
|  |  |  |  |  |  |  |  |  |  |  |  |  |  |  |  |  |  |
| Hung 2021 |  | | | | | |  | | |  | | | Not applicable | | | | |
|  |  |  |  |  |  |  |  |  |  |  |  |  |  |  |  |  |  |
| Kogachi 2023 |  | | | | | |  | | |  | | | Not applicable | | | | |
|  |  |  |  |  |  |  |  |  |  |  |  |  |  |  |  |  |  |
| Koyama 2021 |  | | | | | |  | | |  | | | Not applicable | | | | |
|  |  |  |  |  |  |  |  |  |  |  |  |  |  |  |  |  |  |
| Kuo 2020 |  | | | | | |  | | |  | | | Not applicable | | | | |
|  |  |  |  |  |  |  |  |  |  |  |  |  |  |  |  |  |  |
| Kuo 2021 |  | | | | | |  | | |  | | | Not applicable | | | | |
|  |  |  |  |  |  |  |  |  |  |  |  |  |  |  |  |  |  |
| Kuo 2022 |  | | | | | |  | | |  | | | Not applicable | | | | |
|  |  |  |  |  |  |  |  |  |  |  |  |  |  |  |  |  |  |
| Li 2020 |  | | | | | |  | | |  | | | Not applicable | | | | |
|  |  |  |  |  |  |  |  |  |  |  |  |  |  |  |  |  |  |
| Li 2021 |  | | | | | |  | | |  | | | Not applicable | | | | |
|  |  |  |  |  |  |  |  |  |  |  |  |  |  |  |  |  |  |
| Li 2022 |  | | | | | |  | | |  | | | Not applicable | | | | |
|  |  |  |  |  |  |  |  |  |  |  |  |  |  |  |  |  |  |
| Li 2023 |  | | | | | |  | | |  | | | Not applicable | | | | |
|  |  |  |  |  |  |  |  |  |  |  |  |  |  |  |  |  |  |
| Li 2024 |  | | | | | |  | | |  | | | Not applicable | | | | |
|  |  |  |  |  |  |  |  |  |  |  |  |  |  |  |  |  |  |
| Liang 2023 |  | | | | | |  | | |  | | | Not applicable | | | | |
|  |  |  |  |  |  |  |  |  |  |  |  |  |  |  |  |  |  |
| Liu 2020 |  | | | | | |  | | |  | | | Not applicable | | | | |
|  |  |  |  |  |  |  |  |  |  |  |  |  |  |  |  |  |  |
| Lv 2020 |  | | | | | |  | | |  | | | Not applicable | | | | |
|  |  |  |  |  |  |  |  |  |  |  |  |  |  |  |  |  |  |
| Natarajan 2022 |  | | | | | |  | | |  | | | Not applicable | | | | |
|  |  |  |  |  |  |  |  |  |  |  |  |  |  |  |  |  |  |
| Redd 2022 |  | | | | | |  | | |  | | | Not applicable | | | | |
|  |  |  |  |  |  |  |  |  |  |  |  |  |  |  |  |  |  |
| Soleimani 2023 |  | | | | | |  | | |  | | | Not applicable | | | | |
|  |  |  |  |  |  |  |  |  |  |  |  |  |  |  |  |  |  |
| Tang 2022 |  | | | | | |  | | |  | | | Not applicable | | | | |
|  |  |  |  |  |  |  |  |  |  |  |  |  |  |  |  |  |  |
| Tiwari 2022 |  | | | | | |  | | |  | | | Not applicable | | | | |
|  |  |  |  |  |  |  |  |  |  |  |  |  |  |  |  |  |  |
| Ueno 2024 |  | | | | | |  | | |  | | | Not applicable | | | | |
|  |  |  |  |  |  |  |  |  |  |  |  |  |  |  |  |  |  |
| Wang 2021 |  | | | | | |  | | |  | | | Not applicable | | | | |
|  |  |  |  |  |  |  |  |  |  |  |  |  |  |  |  |  |  |
| Wei 2023 |  | | | | | |  | | |  | | | Not applicable | | | | |
|  |  |  |  |  |  |  |  |  |  |  |  |  |  |  |  |  |  |
| Won 2023 |  | | | | | |  | | |  | | | Not applicable | | | | |
|  |  |  |  |  |  |  |  |  |  |  |  |  |  |  |  |  |  |
| Wu 2018 |  | | | | | |  | | |  | | | Not applicable | | | | |
|  |  |  |  |  |  |  |  |  |  |  |  |  |  |  |  |  |  |
| Wu 2023 |  | | | | | |  | | |  | | | Not applicable | | | | |
|  |  |  |  |  |  |  |  |  |  |  |  |  |  |  |  |  |  |
| Xu 2021 |  | | | | | |  | | |  | | | Not applicable | | | | |
|  |  |  |  |  |  |  |  |  |  |  |  |  |  |  |  |  |  |
| Zhang 2022 |  | | | | | |  | | |  | | | Not applicable | | | | |
|  |  |  |  |  |  |  |  |  |  |  |  |  |  |  |  |  |  |

= Low risk/concern; = Unclear risk/concern; = High risk/concern
