# Supplementary material for: Diagnostic performance of deep learning for infectious keratitis: a systematic review and meta-analysis
Source: eClinicalMedicine. 2024 Oct 18;77:102887. doi: 10.1016/j.eclinm.2024.102887 (PMC11513659; doi:10.1016/j.eclinm.2024.102887)
Supplement: Supplementary Table S3 [file mmc3.docx]

**Supplementary Table 2.** Overview of the diagnostic performance of deep learning (DL) for infectious keratitis (IK), including all internal and external validation/test sets results.

| **Study ID** | **Imaging modality** | **Sensitivity** | **Specificity** |
| --- | --- | --- | --- |
| ***Differentiating IK from non-IK corneal pathologies*** | | | |
| **Internal** | | | |
| Cai 2021 | ASP | 75.0% | NC |
| Li 2020 | ASP | 99.4% | NC |
| Li 2021 | ASP | 97.7% | 98.2% |
| Tiwari 2022 | ASP | 93.5% | 84.4% |
| Ueno 2024 | ASP | 87.8% | 96.9% |
| Huo 2021 | IVCM | 99.3% | 99.2% |
| Liang 2023 | IVCM | 97.0% | 98.5% |
| Liu 2020 | IVCM | 100% | 100% |
| **External (no meta-analyses: < 4 studies after selecting 1 set from each study)** | | | |
| Li 2021_i | ASP | 97.7% | 98.9% |
| Li 2021_ii *Ex. from analyses* | ASP | 97.1% | 97.5% |
| Li 2021_iii *Ex. from analyses* | ASP | 96.8% | 97.0% |
| Li 2021_iv *Ex. from analyses* | ASP | 91.9% | 97.0% |
| Tiwari 2022 | ASP | 78.2% | 91.3% |
| Ueno 2024 | ASP | 76.9% | 99.7% |
| Lv 2020_i | IVCM | 91.9% | 98.3% |
| Lv 2020_ii *Ex. from analyses* | IVCM | 82.6% | 98.9% |
| ***Differentiating causes of IK*** | | | |
| **Internal** | | | |
| Ghosh 2022_i | ASP | 77.0% | 79.8% |
| Ghosh 2022_ii *Ex. from analyses* | ASP | 70.0% | 74.6% |
| Hu 2023 | ASP | 79.2% | 80.1% |
| Hung 2021 | ASP | 66.3% | 87.5% |
| Kuo 2020 | ASP | 71.1% | 68.3% |
| Kuo 2021 | ASP | 82.2% | 54.3% |
| Kuo 2022_i | ASP | 81.3% | 51.6% |
| Kuo 2022_ii *Ex. from analyses* | ASP | 79.7% | 56.5% |
| Li 2022_i | ASP | 73.1% | 70.5% |
| Li 2022_ii *Ex. from analyses* | ASP | 61.7% | 74.0% |
| Li 2022_iii *Ex. from analyses* | ASP | 40.1% | 98.3% |
| Li 2023_i | ASP | 90.5% | 86.0% |
| Li 2023_ii *Ex. from analyses* | ASP | 85.7% | 76.7% |
| Li 2024_i | ASP | 83.5% | 91.7% |
| Li 2024_ii *Ex. from analyses* | ASP | 89.3% | 95.5% |
| Li 2024_iii *Ex. from analyses* | ASP | 79.7% | 96.6% |
| Li 2024_iv *Ex. from analyses* | ASP | 76.9% | 93.8% |
| Li 2024_v *Ex. from analyses* | ASP | 75.0% | 99.9% |
| Natarajan 2022 | ASP | 70.0% | 75.0% |
| Soleimani 2023_i | ASP | 99.4% | 99.2% |
| Soleimani 2023_ii *Ex. from analyses* | ASP | 84.0% | 84.0% |
| Soleimani 2023_iii *Ex. from analyses* | ASP | 77.5% | 76.1% |
| Wei 2023_i | ASP | 98.0% | 74.1% |
| Wei 2023_ii *Ex. from analyses* | ASP | 97.1% | 87.4% |
| Wei 2023_iii *Ex. from analyses* | ASP | 94.8% | 88.1% |
| Won 2023_i | ASP | 87.0% | 88.6% |
| Won 2023_ii *Ex. from analyses* | ASP | 76.1% | 86.3% |
| Wu 2023 | ASP | 91.9% | 83.3% |
| Zhang 2022_i | ASP | 84.1% | 94.7% |
| Zhang 2022_ii *Ex. from analyses* | ASP | 80.0% | 97.9% |
| Zhang 2022_iii *Ex. from analyses* | ASP | 77.8% | 88.0% |
| Zhang 2022_iv *Ex. from analyses* | ASP | 70.5% | 87.1% |
| Essalat 2023_i | IVCM | 99.0% | 99.0% |
| Essalat 2022_ii *Ex. from analyses* | IVCM | 96.0% | 96.5% |
| Essalat 2023_iii *Ex. from analyses* | IVCM | 89.7% | 97.4% |
| Essalat 2023_iv *Ex. from analyses* | IVCM | 91.1% | 98.2% |
| Huang 2022_i | IVCM | 83.2% | 76.6% |
| Huang 2022_ii *Ex. from analyses* | IVCM | 72.0% | 71.6% |
| Tang 2023_i | IVCM | 79.1% | 88.4% |
| Tang 2023_ii *Ex. from analyses* | IVCM | 75.6% | 75.9% |
| Tang 2023_iii *Ex. from analyses* | IVCM | 71.3% | 69.9% |
| Tang 2023_iv *Ex. from analyses* | IVCM | 71.1% | 62.8% |
| Tao 2018 | IVCM | 89.3% | NC |
| Xu 2021 | IVCM | 93.7% | 98.2% |
| **External (no meta-analyses: < 4 studies after selecting 1 set from each study)** | | | |
| Li 2024_i | ASP | 80.5% | 92.6% |
| Li 2024_ii *Ex. from analyses* | ASP | 81.1% | 92.7% |
| Li 2024_iii *Ex. from analyses* | ASP | 80.5% | 95.3% |
| Li 2024_iv *Ex. from analyses* | ASP | 74.8% | 92.7% |
| Li 2024_v *Ex. from analyses* | ASP | 68.3% | 99.4% |
| Wei 2023 | ASP | 90.5% | 90.0% |
| Won 2023_i | ASP | 41.3% | 88.6% |
| Won 2023_ii *Ex. from analyses* | ASP | 32.6% | 81.8% |
| Zhang 2022_i *Ex. from analyses* | ASP | 84.0% | NR |
| Zhang 2022_ii *Ex. from analyses* | ASP | 80.0% | NR |
| Zhang 2022_iii *Ex. from analyses* | ASP | 78.0% | NR |
| Zhang 2022_iv *Ex. from analyses* | ASP | 70.0% | NR |
| **Clinician diagnostic performance** | | | |
| Hu 2023_i | ASP (VK group) | 70.2% | 82.9% |
| Hu 2023_ii *Ex. from analyses* | ASP (FK group) | 60.0% | 85.7% |
| Hu 2023_iii *Ex. from analyses* | ASP (BK group) | 39.3% | 87.3% |
| Kuo 2020_i | ASP (NCS group) | 71.1% | 82.1% |
| Kuo 2020_ii *Ex. from analyses* | ASP (CS group) | 51.6% | 82.7% |
| Kuo 2020_iii *Ex. from analyses* | ASP (Unspecified) | 62.3% | 77.6% |
| Li 2021 | ASP | 96.0% | 97.3% |
| Li 2024_i | ASP (NIK group) | 74.0% | 74.5% |
| Li 2024_ii *Ex. from analyses* | ASP (VK group) | 70.0% | 70.5% |
| Li 2024_iii *Ex. from analyses* | ASP (FK group) | 66.0% | 66.0% |
| Li 2024_iv *Ex. from analyses* | ASP (BK group) | 64.0% | 63.0% |
| Li 2024_v *Ex. from analyses* | ASP (AK group) | 54.0% | 54.5% |
| Redd 2022_i *Ex. from analyses* | ASP (FK group) | 56.3% | 87.5% |
| Redd 2022_ii *Ex. from analyses* | ASP (BK group) | 87.5% | 56.3% |
| Ueno 2024_i | ASP (CS group) | 85.4% | 98.6% |
| Ueno 2024_ii *Ex. from analyses* | ASP (NCS group) | 87.8% | 88.5% |
| Wei 2023 | ASP | 69.1% | 71.4% |
| Xu 2021 | IVCM | 87.0% | 91.7% |
| Zhang 2022_i *Ex. from analyses* | ASP (VK group) | 60.0% | 69.0% |
| Zhang 2022_ii *Ex. from analyses* | ASP (FK group) | 64.0% | 17.0% |
| Zhang 2022_iii *Ex. from analyses* | ASP (BK group) | 48.0% | 31.0% |
| Zhang 2022_iv *Ex. from analyses* | ASP (AK group) | 32.0% | 82.0% |

ASP = Anterior segment photography; IVCM = In vivo confocal microscopy; CS = Corneal specialist; NCS = Non-corneal specialist; VK = Viral keratitis; FK = Fungal keratitis; BK = Bacterial keratitis; AK = Acanthamoeba keratitis; NIK = Non-infectious keratitis; NC = Not calculable; If true negative and false positive was 0 value, “1” is added for analyses

*Ex. from analyses* – Not included in the analyses because: sets with lower sensitivities from studies with multiple test sets, no false positive and true negative data to calculate specificity, or no AI data for comparison with clinician (for Redd 2022 study)
